# Differential Trends in Hookah Use Among New Jersey Youth

**DOI:** 10.5888/pcd16.190097

**Published:** 2019-10-10

**Authors:** Jessica A. Kulak, Michelle T. Bover Manderski, Cristine D. Delnevo, Mary Hrywna, Gregory G. Homish, Gary A. Giovino

**Affiliations:** 1 Department of Health, Nutrition, and Dietetics, Buffalo State College, Buffalo, New York; 2 Center for Tobacco Studies, School of Public Health, Rutgers, The State University of New Jersey, Piscataway, New Jersey; 3 Department of Community Health and Health Behavior, School of Public Health and Health Professions, University at Buffalo, Buffalo, New York

## Abstract

**Introduction:**

Use of 2 or more types of tobacco products is common among youth and young adults, highlighting the need for monitoring and intervention activities to encompass products beyond combustible cigarettes. This study documented patterns and trends of ever, current, and frequent hookah use among high school students in New Jersey by other tobacco product use status.

**Methods:**

We analyzed data from the 2008, 2010, 2012, 2014, 2016 waves of the New Jersey Youth Tobacco Survey. Point estimates and 95% confidence intervals described hookah use stratified by use of other tobacco products. Multivariable logistic regression models assessed trends and correlates of hookah use, controlling for the use of other tobacco products and users’ sociodemographic characteristics. Negative binomial regression models examined the association between total number of tobacco products used and hookah use while controlling for sociodemographic variables and survey year.

**Results:**

The adjusted odds of current and frequent hookah use among New Jersey high school students were significantly higher in 2014, but not in 2016, compared to 2008. In recent years, hookah use among students who had ever smoked hookah, currently smoked hookah, or frequently smoked hookah was more common among students who had ever or currently smoked cigarettes or e-cigarettes. Hookah users consumed a wider variety of other tobacco products than those who did not use hookah.

**Conclusion:**

Hookah use remains a public health concern for adolescents; it is more common among users of other tobacco products, especially cigarette and e-cigarette smokers. Questions remain as to whether users of multiple tobacco products are being adequately reached by existing policies and regulations.

**Summary**

**What is already known on this topic?**

Recent national data on US high school students indicate decreases in current (past 30 day) use of hookah in 2017, following increases in hookah use from 2011 to 2015.

**What is added by this report?**

Hookah use continues to be a public health concern for adolescents, particularly among former and current users of other tobacco products. Analyses of use of combinations of tobacco products are needed because limiting surveillance efforts to single-product use may underestimate nicotine consumption and dependence.

**What are the implications for public health practice?**

Existing public health policies and regulations may not be adequately reaching users of multiple tobacco products.

## Introduction

Alternative tobacco products, including electronic cigarettes (e-cigarettes) and hookah, have increased in popularity in the United States (1). Factors related to the perception of harm (2) and addictive potential (3), the availability of flavored tobacco (4), hookah’s appeal as a social activity (2,3), and general social acceptability (2) can all contribute to the prevalence of hookah use. Recent data on US high school students indicate that current (past 30 days) hookah use declined to 3.3% in 2017 (5), whereas hookah use increased from 4.1% in 2011 to 7.2% in 2015 (6). Data indicate that the increases in adult e-cigarette use occurred primarily among adults who ever (at least once in the lifetime) or currently (in the past 30 days) smoked cigarettes (for purposes of this discussion, “cigarette” refers to conventional, combustible cigarettes): the number of people who had ever used e-cigarettes increased from 2010 to 2014 among current and former cigarette smokers, but not among people who never were cigarette smokers (7,8). An examination of trends in ever and current hookah use (2008–2014) among New York City middle and high school students that included an analysis of hookah use as a function of cigarette smoking status reported that ever hookah use significantly increased among current and former cigarette smokers from 2008 to 2014 (9).

To our knowledge, no peer reviewed articles have reported representative time trend data for hookah use stratified by cigarette smoking status among US adolescents. Data on such patterns of use are vital to understanding their public health implications (10). Use of multiple tobacco products is increasingly common in the modern landscape of nicotine and tobacco products (11) and may increase the prevalence of nicotine dependence (12). In 2017, 9.2% of US high school students used 2 or more tobacco products during the past 30 days (1). Among middle and high school students who used cigarettes, e-cigarettes, cigars, smokeless tobacco, or hookah on at least 20 of the past 30 days, use of multiple tobacco products was reported by over 90% of hookah users (13). Nationally, among current tobacco users aged 18 to 24, 28.4% used 2 or more products in 2017, the highest among current tobacco users of any age group (6). Surveillance data from wave 1 of the Population Assessment of Tobacco and Health Study indicate that 40% of tobacco product users of all ages used at least 2 types of products (14). Tobacco control practitioners have called for the augmentation of monitoring and intervention activities to encompass tobacco products in addition to cigarettes (15), including analyses of use of product combinations (10), because limiting surveillance and resulting analyses to single-product use may underestimate nicotine consumption and dependence.

A limitation of hookah use literature is its focus on current use or use over the past 30 days. Although current use is an important indicator, it conveys only part of the picture and provides no details on product initiation or amount of use. Information on patterns of tobacco product use is important for informing tobacco prevention and control activities (13). Measurements of lifetime use can provide data on product experimentation, whereas measurement of the frequency of hookah use provides data on amount of use and number of days used. Therefore, a more nuanced monitoring of product use across multiple indicators is needed. Our study explores trends and patterns over 9 years among New Jersey high school students for 3 indicators of hookah use — ever, current, and frequent use — as a function of other tobacco products used.

## Methods

### Data source

The New Jersey Youth Tobacco Survey (NJYTS) is a cross-sectional survey that has been administered roughly every 2 years to New Jersey high school students (grades 9–12) since 1999. Questions about hookah were added to the survey in 2008. We used data from waves 2008, 2010, 2012, 2014 and 2016 of the survey for this article. The survey methodology (16) was approved by the Health Sciences Institutional Review Board at Rutgers, the State University of New Jersey. The study sampled 3,042 students in 2008, 2,641 students in 2010, 1,850 in 2012, 3,909 in 2014, and 3,604 in 2016. Overall participation rates ranged from 60% to 81%, and data were weighted by the Centers for Disease Control and Prevention (16).

### Measures

The 3 indicators of hookah use were the dependent variables of ever hookah use (use at least once during the lifetime), current hookah use (any use during the past 30 days), and frequent hookah use. Frequent hookah use was measured in days used in the past month; a binary variable was created, where frequent hookah use was use on 10 or more days per month, a definition consistent with previous research (16).

Other variables included never, former, and current cigarette smoking. Here, ever is defined as smoking a cigarette at least once during the lifetime; former is cigarette smoking within the lifetime, but not in the past 30 days; and current is defined as smoking on at least one day in the past 30 days. Other tobacco products of interest were cigars, smokeless tobacco, and bidis (measured 2008–2016), and e-cigarettes, snus, pipes, and dissolvable tobacco (measured 2012–2016). For each of these products, ever is defined as use of the product at least once during the lifetime, whereas current is defined as use of the product on at least 1 of the past 30 days.

### Statistical analysis

Data were analyzed by using Stata, version 14.2 (StataCorp LLC). All analyses were weighted and used a Taylor series linearization method to account for the complex sampling design of the NJYTS. Point estimates and 95% confidence intervals (CIs) were calculated by using all the data from each survey year for ever hookah use, current hookah use, and frequent hookah use. All surveys provided data demographically representative of public high school students in New Jersey.

Three multivariable logistic regression analyses (one each for ever hookah use, current hookah use, and frequent hookah use) were conducted by using 2008–2016 combined data to produce adjusted odds ratios for each of these dependent variables. Models controlled for sociodemographics (sex, race/ethnicity, grade), survey year, and use of other tobacco products. Finally, negative binomial regression models examined the relationship between total number of tobacco products used (a count variable, excluding hookah, with a range from 0 to 8) and ever, current, and frequent hookah use, controlling for sociodemographic characteristics and survey year. These models were restricted to 2012–2016 because measures of e-cigarettes, snus, pipes, and dissolvable tobacco were added to the survey in 2012.

## Results

**Ever hookah use.** In 2016, 15.9% of New Jersey high school students had ever used a hookah (Table 1). By comparison, 21.0% of students in 2016 had ever tried e-cigarettes, 17.2% had tried cigars, and 16.4% had tried cigarettes. Compared with 2008, the adjusted odds of ever using a hookah were significantly higher in 2012 (aOR, 1.45; 95% CI, 1.04–2.02), 2014 (aOR, 2.46; 95% CI, 1.88–3.22), and 2016 (aOR, 1.54; 95% CI, 1.15–2.07) (Table 2). Ever and current cigarette, cigar, and bidi smokers and ever and current smokeless tobacco users all had greater odds of ever hookah use than did students who had never used those products (Table 2). When data on e-cigarettes, regular pipes, snus, and dissolvable tobacco products were available (2012–2016) (Table 3), the odds of ever using a hookah were elevated among ever and current cigarette smokers and users of e-cigarettes, cigars, and the combined indicator of regular pipes, snus, and dissolvable tobacco products.

**Table 1:** Hookah Use Among New Jersey High School Students, by Cigarette Use, New Jersey Youth Tobacco Survey, 2008–2016^a^

Hookah Use^b^	2008, N = 3,042	2010, N = 2,641	2012, N = 1,850	2014, N = 3,909	2016, N = 3,604
**Overall**					
Ever	17.8 (15.4–20.5)	20.9 (18.3–23.8)	18.5 (15.4–22.1)	23.6 (20.8–26.7)	15.9 (12.9–19.5)
Currently	9.6 (8.1–11.4)	11.4 (9.8–13.3)	8.4 (6.6–10.6)	11.8 (10.1–13.7)	7.0 (5.0–9.6)
Frequently	1.6 (1.2–2.1)	1.3 (0.8–2.2)	1.4 (0.9–2.3)	2.9 (2.2–3.8)	1.6 (1.0–2.5)
**Never smoked cigarettes**					
Ever	8.6 (6.5–11.4)	9.8 (7.6–12.6)	6.2 (4.2–8.9)	11.3 (9.2–13.7)	8.9 (6.8–11.7)
Currently	4.7 (3.4–6.6)	5.3 (3.6–7.7)	2.4 (1.4–3.8)	4.3 (3.4–5.4)	2.6 (1.8–3.8)
Frequently	0.5 (0.2–1.1)	0.7 (0.3–1.4)	0.1 (0.0–0.5)	0.7 (0.4–1.1)	0.3 (0.1–0.9)
**Former cigarette smoker**					
Ever	28.7 (24.8–32.9)	32.5 (25.2–40.8)	38.3 (29.4–48.2)	51.7 (46.3–57.0)	41.3 (36.1–46.8)
Currently	11.4 (8.9–14.5)	14.4 (12.5–16.6)	11.8 (8.4–16.5)	19.7 (16.0–23.9)	12.6 (7.9–19.6)
Frequently	1.4 (0.8–2.5)	1.3 (0.5–3.0)	2.0 (0.8–4.8)	4.2 (3.0–5.8)	2.1 (1.1–4.0)
**Current cigarette smoker**					
Ever	45.6 (39.7–51.8)	59.8 (52.4–66.7)	67.4 (57.5–76.0)	72.7 (66.0–78.4)	58.6 (49.5–67.2)
Currently	28.7 (23.6–34.4)	36.0 (28.4–44.4)	41.3 (30.7–52.7)	51.3 (44.8–57.7)	36.1 (28.0–45.1)
Frequently	5.9 (3.9–8.7)	3.9 (2.1–7.4)	8.5 (4.7–14.8)	16.4 (10.9–24.1)	14.3 (8.5–22.9)

a Values are percentage (95% confidence interval).

b Ever is defined as hookah use at least once during the lifetime; currently is defined as hookah use within the past 30 days; frequently is defined as hookah use on 10 or more days per month.

**Table 2:** Multivariable Logistic Regression Analysis of Hookah Use Among New Jersey High School Students, by Other Tobacco Product Use, Demographic Characteristics, and Year, New Jersey Youth Tobacco Survey, 2008–2016^a^

Variable^b^	Ever Hookah Use^b^^,^ c	Current Hookah Use^b^^,^ c	Frequent Hookah Use^b^^,^ c
**Use of Other Tobacco Products**			
**Cigarette**			
Never	9.0 (1.00) [reference]	3.8 (1.00) [reference]	0.4 (1.00) [reference]
Ever, not currently	38.0 (3.69) [3.09–4.41]^d^	14.1 (2.58) [2.08–3.21]^d^	2.2 (2.65) [1.62–4.36]^d^
Currently, 1–9 DPM	55.2 (5.93) [4.65–7.55]^d^	34.3 (5.65) [4.17–7.66]^d^	5.9 (3.87) [1.74–8.63]^d^
Currently, ≥10 DPM	67.3 (6.54) [4.74–9.01]^d^	43.5 (4.81) [3.19–7.27]^d^	13.0 (5.99) [2.82–12.69]^d^
**Cigar**			
Never	11.2 (1.00) [reference]	5.0 (1.00) [reference]	0.6 (1.00) [reference]
Ever, not currently	42.3 (2.51) [2.16–2.92]^d^	12.8 (1.24) [0.98–1.56]	2.0 (1.26) [0.74–2.16]
Currently, 1-9 DPM	55.8 (2.94) [2.24–3.86]^d^	39.7 (2.99) [2.14–4.18]^d^	7.2 (1.93) [1.07–3.47]^d^
Currently, ≥10 DPM	63.0 (2.48) [4.45–4.23]^d^	63.4 (3.39) [1.96–5.87]^d^	29.5 (4.47) [2.12–9.43]^d^
**Smokeless tobacco**			
Never	15.4 (1.00) [reference]	6.4 (1.00) [reference]	0.8 (1.00) [reference]
Ever, not currently	46.6 (1.93) [1.58–2.36]^d^	17.0 (1.61) [1.15–2.26]^d^	2.1 (1.34) [0.68–2.64]
Currently, 1–9 DPM	54.8 (1.72) [1.21–2.45]^d^	47.1 (3.36) [2.26–5.01]^d^	11.6 (3.34) [1.78–6.26]^d^
Currently, ≥10 DPM	67.9 (2.18) [1.20–3.95]^d^	56.2 (2.96) [1.65–5.32]^d^	24.6 (3.12) [1.64–5.89]^d^
**Bidi**			
Never	15.0 (1.00) [reference]	6.0 (1.00) [reference]	0.8 (1.00) [reference]
Ever, not currently	58.1 (3.86) [3.01–4.96]^d^	15.0 (1.02) [0.75–1.38]	2.3 (0.77) [0.29–2.09]
Currently, 1–9 DPM	61.6 (7.72) [5.24–11.39]^d^	62.4 (10.7) [7.50–15.36]^d^	10.6 (2.69) [1.53–4.72]^d^
Currently, ≥10 DPM	58.8 (1.80) [0.71–4.58]	81.1 (9.64) [3.81–24.39]^d^	48.7 (15.8) [7.87–31.96]^d^
**Demographic Characteristic**			
**Sex**			
Male	19.4 (1.00) [reference]	10.0 (1.00) [reference]	2.2 (1.00) [reference]
Female	19.4 (1.49) [1.32–1.67]^d^	9.2 (1.67) [1.38–2.01]^d^	1.3 (1.09) [0.76–1.57]
**Grade**			
9	12.1 (1.00) [reference]	7.0 (1.00) [reference]	1.2 (1.00) [reference]
10	16.8 (1.49) [1.21–1.84]^d^	9.4 (1.19) [0.88–1.63]	1.8 (1.02) [0.62–1.68]
11	21.2 (1.55) [1.30–1.85]^d^	10.6 (1.22) [0.96–1.54]	1.6 (1.00) [0.63–1.61]
12	28.2 (2.12) [1.72–2.62]^d^	11.5 (1.12) [0.85–1.48]	2.5 (1.50) [0.92–2.47]
**Race/ethnicity**			
Non-Hispanic white	18.8 (1.00) [reference]	8.0 (1.00) [reference]	1.3 (1.00) [reference]
Non-Hispanic black	16.9 (0.95) [0.77–1.18]	10.5 (1.28) [0.92–1.80]	2.2 (1.99) [1.12–3.52]^d^
Hispanic	24.5 (1.52) [1.27–1.81]^d^	13.9 (2.10) [1.67–2.65]^d^	2.8 (2.41) [1.53–3.80]^d^
Non-Hispanic other	15.9 (1.12) [0.90–1.40]	8.1 (1.46) [1.06–2.01]^d^	1.5 (1.81) [0.96–3.41]
**New Jersey Youth Tobacco Survey Year**			
2008	17.8 (1.00) [reference]	9.6 (1.00) [reference]	1.6 (1.00) [reference]
2010	20.9 (1.36) [0.98–1.89]	11.4 (1.27) [0.91–1.77]	1.3 (0.80) [0.44–1.46]
2012	18.5 (1.45) [1.04–2.02]^d^	8.4 (1.27) [0.86–1.89]	1.4 (1.12) [0.68–1.84]
2014	23.6 (2.46) [1.88–3.22]^d^	11.8 (2.01) [1.47–2.76]^d^	2.9 (2.68) [1.74–4.13]^d^
2016	15.9 (1.54) [1.15–2.07]^d^	7.0 (0.99) [0.67–1.47]	1.6 (1.46) [0.88–2.44]

Abbreviations: AOR, adjusted odds ratio; CI, confidence interval; DPM, days per month.

a Model controls for all other variables in table except for e-cigarettes, pipes, dissolvable tobacco, and snus, because they were not measured in all survey waves.

b Values are percentage (adjusted odds ratio) [confidence interval].

c Ever hookah use was defined as smoking hookah at least once in the lifetime, current use as smoking hookah within the past 30 days, and frequent use as smoking hookah on more than 10 DPM. Ever use was determined by asking, “Have you ever used a hookah to smoke tobacco or flavored tobacco?” Current and frequent hookah use were determined by asking, “During the past 30 days, on how many days did you use a hookah to smoke tobacco or flavored tobacco?”

d Significant at *P* < .05.

**Table 3:** Multivariable Logistic Regression Analysis of Hookah Use Among New Jersey High School Students, by Other Tobacco Product Use, Demographic Characteristics, and Year, New Jersey Youth Tobacco Survey, 2012–2016

Variable	Ever Hookah Use^a^^,^ b ^,^ c	Current Hookah Use^a^^,^ b ^,^ c	Frequent Hookah Use^a^^,^ b ^,^ c
**Use of Other Nicotine/Tobacco Product**			
**Cigarette**			
Never	9.0 (1.00) [Reference]	3.8 (1.00) [Reference]	0.4 (1.00) [Reference]
Ever, not currently	38.0 (3.08) [2.46–3.86]^d^	14.1 (2.29) [1.70–3.08]^d^	2.2 (2.79) [1.42–5.50]^d^
Currently, 1–9 DPM	55.2 (3.77) [2.61–5.47]^d^	34.3 (4.33) [2.95–6.36]^d^	5.9 (3.16) [1.10–9.09]^d^
Currently, ≥10 DPM	67.3 (3.63) [2.22–5.93]^d^	43.5 (2.38) [1.27–4.46]^d^	13.0 (3.80) [1.25–11.62]^d^
**E-cigarette**			
Never	10.3 (1.00) [Reference]	4.0 (1.00) [Reference]	0.5 (1.00) [Reference]
Ever, not currently	48.7 (2.97) [2.36–3.74]^d^	10.9 (1.14) [0.73–1.78]	2.5 (2.22) [1.04–4.76]^d^
Currently, 1-9 DPM	57.6 (4.29) [3.16–5.82]^d^	44.4 (5.64) [3.90–8.16]^d^	7.8 (2.83) [1.22–6.57]^d^
Currently, ≥10 DPM	70.5 (5.79) [3.59–9.35]^d^	63.9 (7.10) [4.05–12.45]^d^	32.5 (12.38) [5.47–28.05]^d^
**Cigar**			
Never	11.2 (1.00) [Reference]	5.0 (1.00) [Reference]	0.6 (1.00) [Reference]
Ever, not currently	42.3 (2.29) [1.80–2.91]^d^	12.8 (1.01) [0.73–1.40]	2.0 (0.74) [0.39–1.40]
Currently, 1-9 DPM	55.8 (2.08) [1.44–3.00]^d^	39.7 (2.01) [1.20–3.38]^d^	7.2 (1.07) [0.57–2.01]
Currently, ≥10 DPM	63.0 (1.88) [1.01–3.48]^d^	63.4 (2.08) [1.00–4.32]	29.5 (1.70) [0.73–3.98]
**Smokeless tobacco**			
Never	15.4 (1.00) [Reference]	6.4 (1.00) [Reference]	0.8 (1.00) [Reference]
Ever , not currently	46.6 (1.61) [1.10–2.35]^d^	17.0 (1.25) [0.72–2.17]	2.1 (1.35) [0.61–3.00]
Currently, 1–9 DPM	54.8 (0.90) [0.45–1.78]	47.1 (1.63) [0.79–3.40]	11.6 (2.50) [1.01–6.22]^d^
Currently, ≥10 DPM	67.9 (1.22) [0.46–3.23]	56.2 (1.83) [0.69–4.85]	24.6 (1.68) [0.65–4.34]
**Bidi**			
Never	15.0 (1.00) [Reference]	6.0 (1.00) [Reference]	0.8 (1.00) [Reference]
Ever, not currently	58.1 (1.33) [0.94–1.87]	15.0 (0.69) [0.45–1.07]	2.3 (0.68) [0.26–1.76]
Currently, 1–9 DPM	61.6 (1.18) [0.68–2.07]	62.4 (2.76) [1.44–5.28]^d^	10.6 (0.62) [0.28–1.37]
Currently, ≥10 DPM	58.8 (0.28) [0.11–0.72]	81.1 (2.62) [0.91–7.55]	48.7 (3.88) [1.51–10.01]^d^
**Other tobacco products^e^**			
Never	11.9 (1.00) [Reference]	4.1 (1.00) [Reference]	0.5 (1.00) [Reference]
Ever, not currently	60.5 (5.04) [3.85–6.60]^d^	11.3 (1.15) [0.75–1.76]	2.3 (1.65) [0.64–4.25]
Current user	64.6 (5.97) [4.03–8.84]^d^	68.2 (10.78) [7.30–15.90]^d^	20.6 (6.02) [2.85–12.71]^d^
**Demographic Characteristics**			
**Sex**			
Male	19.4 (1.00) [Reference]	10.0 (1.00) [Reference]	2.2 (1.00) [Reference]
Female	19.4 (1.94) [1.62–2.32]^d^	9.2 (2.12) [1.56–2.89]^d^	1.3 (1.11) [0.67–1.84]
**Grade**			
9	12.1 (1.00) [Reference]	7.0 (1.00) [Reference]	1.2 (1.00) [Reference]
10	16.8 (1.82) [1.39–2.38]^d^	9.4 (1.54) [0.99–2.40]	1.8 (0.89) [0.46–1.71]
11	21.2 (1.81) [1.44–2.68]^d^	10.6 (2.10) [1.59–2.79]^d^	1.6 (1.19) [0.62–2.30]
12	28.2 (2.38) [1.82–3.12]^d^	11.5 (1.54) [1.07–2.22]^d^	2.5 (1.40) [0.76–2.59]
**Race/ethnicity**			
Non-Hispanic white	18.8 (1.00) [Reference]	8.0 (1.00) [Reference]	1.3 (1.00) [Reference]
Non-Hispanic black	16.9 (1.28) [0.97–1.70]	10.5 (1.75) [1.17–2.60]^d^	2.2 (2.40) [1.10–5.22]^d^
Hispanic	24.5 (1.85) [1.51–2.27]^d^	13.9 (2.96) [2.21–3.97]^d^	2.8 (2.84) [1.64–4.92]^d^
Non-Hispanic other	15.9 (1.14) [0.83–1.58]	8.1 (1.58) [0.98–2.55]	1.5 (1.65) [0.70–3.90]
**New Jersey Youth Tobacco Survey**			
2012	18.5 (1.00) [Reference]	8.4 (1.00) [Reference]	1.4 (1.00) [Reference]
2014	23.6 (1.40) [0.99–1.96]	11.8 (1.31) [0.88–1.95]	2.9 (2.22) [1.23–4.00]^d^
2016	15.9 (0.86) [0.61–1.23]	7.0 (0.69) [0.43–1.11]	1.6 (1.35) [0.69–2.67]

Abbreviations: AOR, adjusted odds ratio; CI, confidence interval; DPM, days per month.

a Model controls for all other variables in table except for e-cigarettes, pipes, dissolvable tobacco, and snus, because these products were not measured in all survey waves.

b Values are percentage (adjusted odds ratio) [confidence interval].

c Ever hookah use was defined as smoking hookah at least once in the lifetime, current use, as smoking hookah within the past 30 days, and frequent use as smoking hookah on more than 10 DPM. Ever use was determined by asking, “Have you ever used a hookah to smoke tobacco or flavored tobacco?” Current and frequent hookah use were determined by asking, “During the past 30 days, on how many days did you use a hookah to smoke tobacco or flavored tobacco?”

d Significant at *P* < .05.

e Includes pipes, dissolvable tobacco, and snus. The New Jersey Youth Tobacco Survey measured these products only in yearly surveys for 2012 through 2016.

**Current hookah use.** In 2016, 7.0% of all New Jersey high school students reported using a hookah during the previous 30 days (Table 1) compared with 9.5% for e-cigarettes, 6.8% for cigars, and 4.8% for cigarettes. Compared with 2008, odds of current hookah use were higher in 2014 (aOR, 2.01; 95% CI, 1.47–2.76), though not significantly different in 2016 (aOR, 0.99; 95% CI, 0.67–1.47) (Table 2). In multivariable models, odds of current e-cigarette use increased significantly in 2014 (aOR, 2.23; 95% CI, 1.50–3.31) and 2016 (aOR, 2.13; 95% CI, 1.21–3.74) compared with 2012. During 2008–2016, the adjusted odds of current hookah use were higher compared with never smokers among current and ever cigarette smokers, current and ever smokeless tobacco users, and current cigar and bidi smokers (Table 2). During 2012–2016, the odds of currently using hookah were elevated among ever and current cigarette smokers and among current smokers/users of e-cigarettes, cigars, bidis, and the combined indicator of regular pipes, snus, and dissolvable tobacco products (Table 3).

**Frequent hookah use.** Among all students, frequent hookah use was low from 2008 (1.6%) to 2016 (1.6%) (Table 1). Compared with 2008, odds of frequent hookah use were significantly higher in 2014 (aOR, 2.68; 95% CI, 1.74–4.13) but not in 2016 (aOR, 1.46; 95% CI, 0.88–2.44) (Table 2). The odds of frequent hookah use were significantly higher for users of other tobacco products, including ever and current combustible cigarette smokers, current cigar and bidi smokers, and current smokeless tobacco users (Table 2). During 2012–2016, the odds of frequently using hookah were elevated among ever and current smokers of cigarettes, ever and current users of e-cigarettes, current (1–9 days/month) smokeless tobacco users, current (≥10 days/month) bidi smokers, and current smokers/users of the combined indicator of regular pipes, snus, and dissolvable tobacco products (Table 3).

**Hookah use as a function of the number of other tobacco products currently used.** Hookah was commonly used in combination with other products (Table 4). Although 26.2% of current hookah users reported using no other products, 23% used at least 1 additional product and more than half (51.1%) used 2 or more products in addition to hookah. Finally, in negative binomial regression models controlling for sociodemographic variables and survey year, ever (β, 1.90; 95% CI, 1.74–2.05), current (β, 2.66; 95% CI, 2.52–2.81), and frequent hookah use (β, 2.37; 95% CI, 2.20–2.54) showed a significantly higher expected number of tobacco products used among ever, current, and/or frequent hookah users compared with nonusers.

**Table 4:** Current^a^ Hookah Use Among New Jersey High School Students, by Concurrent Use of Other Tobacco Products, by Year, New Jersey Youth Tobacco Survey, 2008–2016

Tobacco Product	Current Hookah Use^b^				
	2008	2010	2012	2014	2016
Cigarette	28.7 (23.6–34.4)	36.0 (28.4–44.4)	41.3 (30.7–52.7)	51.3 (44.8–57.7)	36.1 (29.1–43.8)
Cigar	39.5 (33.1–46.4)	49.2 (42.1–56.3)	36.2 (25.8–48.0)	53.3 (46.0–60.5)	47.1 (36.6–57.8)
E-cigarette	—	—	57.1 (45.2–68.3)	53.1 (47.5–58.6)	39.7 (31.5–48.6)
Bidi	55.6 (48.9–62.2)	62.8 (54.7–70.2)	75.3 (55.1–88.3)	79.3 (71.8–85.2)	72.4 (66.1–78.0)
Smokeless	46.8 (36.6–57.4)	41.5 (29.4–54.8)	53.5 (37.0–69.2)	59.0 (50.1–67.3)	53.1 (40.6–65.2)
Snus	—	—	71.1 (48.7–86.5)	81.3 (71.9–88.1)	70.8 (60.9–79.0)
Pipe	—	—	75.2 (63.0–84.3)	77.6 (70.5–83.4)	72.6 (59.0–83.0)
Dissolvable tobacco	—	—	67.3 (51.3–80.2)	77.8 (68.3–85.0)	66.6 (56.5–75.3)
Any tobacco product	38.7 (34.3–43.2)	45.9 (40.0–52.0)	44.9 (35.8–54.4)	51.5 (46.6–56.3)	40.6 (33.0–48.7)

Abbreviation: —, not available.

a Current use is defined as use within the past 30 days.

b Values are weighted percentage (95% confidence interval).

## Discussion

Prevalence of hookah use and trends varied considerably by lifetime history of cigarette smoking and by use of other nicotine and tobacco products. The general pattern of increased hookah use among former and current cigarette smokers was consistent for ever, current, and frequent hookah use. Even in 2016, when hookah use decreased, hookah prevalence was still significantly greater among former and current cigarette smokers than among never smokers. Similar to our findings, data from New York City youth demonstrate significant increases over time in ever hookah use, and report nonsignificant increases in current hookah use among current cigarette smokers (9).

Our study provides detailed analyses of adolescent hookah use as a function of cigarette smoking status while considering the greater tobacco product landscape. The study complements nationally available data on hookah use trends by also including data for ever and frequent hookah use and by demonstrating that increases in hookah use appear to be driven by students who have also tried or currently use cigarettes or other tobacco products. Our results support the findings from New York City that hookah use among youth occurs more commonly among cigarette smokers, and we extend their findings by demonstrating that use of multiple tobacco products is common among hookah users; nearly one-quarter (22.7%) of current hookah users used at least 1 additional product, and more than half (51.1%) used 2 or more products in addition to hookah.

Trend data for ever hookah use provide detail on product experimentation, whereas frequent hookah use provides information on amount of use, constructs that can inform tobacco control practitioners. Trends in these 2 indicators mirror those of current hookah use in both New Jersey (17) and at the national level (5,18) for all 3 indicators (ever, current, and frequent use); hookah use appears to have peaked in 2014 and has decreased since. This downward trend, observed across all 3 measures and among other tobacco product users, is encouraging, because it may represent a true turning point in the hookah use epidemic (18).

Numerous socio-environmental factors may have contributed to the increase and subsequent decrease of hookah use. In 2012, only 41% of youth reported awareness of hookah (19); by 2014, hookah was the leading combustible tobacco product currently used by US middle and high school students (6). Surges in hookah’s popularity may be attributed to the rise in the hookah café environment from 2000 through 2010 (20), which may have been the inadvertent result of implementation of smoke-free laws and policies and loopholes in regulations (20). These loopholes persist despite the fact that the air quality in hookah bars often exceeds established air quality standards, and levels of particulate matter and carbon monoxide are often higher than what is seen in bars where cigarette smoking is still allowed (21). Furthermore, hookah lounges and retailers are not monitored or controlled in the same way as cigarette product retailers (22).

Socio-environmental factors associated with the online presence of tobacco products may also have contributed to an increased prevalence of hookah use in the last decade. The internet has extended the exposure and reach for all types of tobacco and nicotine products (23). Hookah has been prominently featured on social media, including Twitter (24), Instagram (25), and Facebook (26), by individuals (24,25), businesses (24,25), and by product-affiliated brands (26). Social media content is largely positive (24), and advertising and promotions exist even on social media platforms that have policies against the promotion and sale of tobacco products (26). Few social media posts mention health consequences of hookah use (25), possibly perpetuating misconceptions that hookah is safer than other tobacco products. An improved understanding is needed of the influence of internet advertisements and social media–based marketing of single and multiple tobacco products on adolescent tobacco use.

Recent decreases in hookah use may be an indicator that hookah has become a more prominent focus of public policy and regulation since the early-to-mid 2010s. Most notably, an amendment to the Family Smoking Prevention and Tobacco Control Act was proposed in 2014, recommending hookah tobacco be included under the US Food and Drug Administration’s (FDA) authority (27). The amendment was finalized in 2016, and hookah products now must meet FDA regulations for packaging and labeling, health warning statements, and the disclosure of product constituents (28). Furthermore, sales of hookah are restricted to people aged 18 or older, and photo identification is required of anyone who appears younger than 27 (28). Although the FDA amendment was finalized after the data for our study were collected, it was originally proposed in 2014 (27); this original proposal may have generated a focus on tobacco control efforts aimed at hookah products.

Flavored hookah tobacco is popular among both youth and adults (3), and the availability of characterizing flavors in these products is often cited as a reason for hookah use (4). Currently, the FDA’s deeming rule does not ban flavors in hookah tobacco products (28). However, the FDA announced in March 2019 an advance notice of proposed rulemaking to obtain information and inform regulatory action on the role that flavors play in tobacco products, including hookah (29). Flavored tobacco use is high among hookah users and is associated with use of multiple tobacco products and fewer quit attempts (30). State and local governments have begun implementing laws restricting flavors in tobacco products. Various municipalities have passed laws regulating the sale of flavored tobacco products (31), which may have helped in more recent reductions in initiation of hookah use (30). More research on the effect of these bans will be needed to determine their long-term effect on hookah use.

Our study had limitations. Notably, the number of respondents in the 2012 wave was limited because Hurricane Sandy hit New Jersey during the data collection phase, resulting in a lower overall response rate and wider confidence intervals. However, 2012 estimates do not differ statistically from those of 2010. Furthermore, New Jersey data are not nationally representative, though data from this state have been mirroring national trends among US high school students (5,6,17,18). These data are based on self-report. Given that youth may be confused by the terminology used to describe specific products (32), errors may have occurred in their reporting of use of nonconventional tobacco products. Finally, among ever users, initiation could have happened a long time ago. Age of first use is not captured by these data. Despite these limitations, we purposefully selected this state’s data because it allows for nuanced analyses of hookah trends, whereas the YTS does not, because it did not include data on frequent use until 2016. In the NJYTS, hookah use was queried consistently with 2 forced-choice questions during the 2008–2016 waves. Future research may be needed to replicate these findings by using a nationally representative sample.

In summary, hookah use was more concentrated among current cigarette smokers and users of other tobacco products (including e-cigarette users) than among never and former tobacco product users. These data provide additional information needed for a comprehensive effort to control youth tobacco use. Despite overall declining proportions of youth use, hookah use continues to be a public health concern for adolescents, particularly among former and current cigarette smokers. Questions remain as to whether multiple-product users are being adequately reached by existing policies and regulations. Longitudinal studies such as the Population Assessment of Tobacco and Health study (33) that examine dual product use in the context of the current regulatory environment are necessary. Numerous underused regulatory avenues exist for improving policies governing hookah use, including regulations of flavors (29). Comprehensive, systematic approaches to tobacco control, that take into account both individual and socioenvironmental factors (34), are necessary to stem trial, initiation, and maintenance of the use of all tobacco products among US youth.
